# Distinctive patterns of sulfide- and butyrate-metabolizing bacteria after bariatric surgery: potential implications for colorectal cancer risk

**DOI:** 10.1080/19490976.2023.2255345

**Published:** 2023-09-13

**Authors:** Hisham Hussan, Steven K. Clinton, Elizabeth M. Grainger, Maxine Webb, Cankun Wang, Amy Webb, Bradley Needleman, Sabrena Noria, Jiangjiang Zhu, Fouad Choueiry, Maciej Pietrzak, Michael T. Bailey

**Affiliations:** a Division of Gastroenterology, Department of Internal Medicine, University of California, Davis; Sacramento, CA, USA; b The UC Davis Comprehensive Cancer Center, Sacramento, CA, USA; c Division of Medical Oncology; Department of Internal Medicine, The Ohio StateUniversity, Columbus, OH, USA; d The Ohio State University Comprehensive Cancer Center, Columbus, OH, USA; e Division of Biomedical Informatics, Department of Biomedical Informatics, The Ohio State University, Columbus, OH, USA; f Center for Minimally Invasive Surgery; Department of General Surgery, The Ohio State University, Columbus, OH, USA; g The Department of Human Sciences, The Ohio State University, Columbus, OH, USA; h Center for Microbial Pathogenesis, Abigail Wexner Research Institute at Nationwide Children’s Hospital and Department of Pediatrics, Columbus, OH, USA; i The Oral and Gastrointestinal Microbiology Research Affinity Group, Abigail Wexner Research Institute at Nationwide Children’s Hospital, Columbus, OH, USA

**Keywords:** Bariatric, microbiome, sulfide-reducing, butyrate, colorectal cancer

## Abstract

Despite improved cardiometabolic outcomes following bariatric surgery, its long-term impact on colorectal cancer (CRC) risk remains uncertain. In parallel, the influence of bariatric surgery on the host microbiome and relationships with disease outcomes is beginning to be appreciated. Therefore, we investigated the impact of Roux-en-Y gastric bypass (RYGB) and vertical sleeve gastrectomy (VSG) on the patterns of sulfide-reducing and butyrate-producing bacteria, which are hypothesized to modulate CRC risk after bariatric surgery. In this single-center, cross-sectional study, we included 15 pre-surgery subjects with severe obesity and patients who are at a median (range) of 25.6 (9.9–46.5) months after RYGB (*n* = 16) or VSG (*n* = 10). The DNA abundance of fecal bacteria and enzymes involved in butyrate and sulfide metabolism were identified using metagenomic sequencing. Differences between pre-surgery and post-RYGB or post-VSG cohorts were quantified using the linear discriminant analysis (LDA) effect size (LEfSe) method. Our sample was predominantly female (87%) with a median (range) age of 46 (23–71) years. Post-RYGB and post-VSG patients had a higher DNA abundance of fecal sulfide-reducing bacteria than pre-surgery controls (LDA = 1.3–4.4, *p* < .05). The most significant enrichments were for fecal *E. coli*, *Acidaminococcus* and *A. finegoldii* after RYGB, and for *A. finegoldii*, *S. vestibularis, V. parvula* after VSG. As for butyrate-producing bacteria, *R. faecis* was more abundant, whereas *B. dentium* and *A. hardus* were lower post-RYGB vs. pre-surgery. *B. dentium* was also lower in post-VSG vs. pre-surgery. Consistent with these findings, our analysis showed a greater enrichment of sulfide-reducing enzymes after bariatric surgery, especially RYGB, vs. pre-surgery. The DNA abundance of butyrate-producing enzymes was lower post-RYGB. In conclusion, the two most used bariatric surgeries, RYGB and VSG, are associated with microbiome patterns that are potentially implicated in CRC risk. Future studies are needed to validate and understand the impact of these microbiome changes on CRC risk after bariatric surgery.

## Introduction

Fifty percent of Americans will be classified as having obesity, and 25% will have severe obesity (BMI ≥35 kg/m^2^) by 2030.^1^ The risk of colorectal cancer (CRC) is increased by 30% with rising BMI.^2^ Therefore, an impactful weight-loss management ranging from behavior modification, novel pharmaceuticals, and surgical approaches such as Roux-en-Y gastric bypass (RYGB) and vertical sleeve gastrectomy (VSG), should hypothetically reduce the subsequent risk of CRC. Given the health risks associated with severe obesity, surgical approaches are increasingly being employed in these individuals.^3^ Indeed, many immune and metabolic cancer-promoting biological processes are improved after bariatric surgery, including a marked reduction in biomarkers of systemic inflammation and insulin resistance.^4–9^ However, the impact of bariatric surgery on colorectal cancer risk remains unclear. For instance, short-term studies report a reduction in CRC risk that is limited to females within 10 years after bariatric surgery, despite a more pronounced weight loss in males post-surgery.^10–12^ Studies with longer follow-up durations report no impact or increased CRC risk in males and females 10 years after surgery.^13–15^ Human observational and animal mechanistic studies also suggest a mixed effect of bariatric surgery on colorectal inflammatory cascades and biomarkers of carcinogenesis.^16–20^ Since progression from normal tissue to CRC can take 10–15 years, a thorough understanding of the impact of bariatric surgery on the colorectal milieu is warranted to improve our understanding of CRC risk in this rapidly expanding population.^21^

One area that remains poorly understood is how bariatric surgery alters the colonic microbiome and its implications for CRC risk. Indeed, evolving evidence supports the important role of the colonic microbiome in CRC development, although more research is warranted to establish firm causative links. Specifically, sulfide-reducing bacteria are increasingly identified as possible contributors to distal colorectal cancer risk.^22,23^ It is hypothesized that sulfide-reducing bacteria promote carcinogenesis by reducing dietary sulfur to hydrogen sulfide through multiple routes.^24^ Hydrogen sulfide promotes inflammation, increased DNA damage, and disrupts of the protective colonic mucus layer.^25–27^ Conversely, butyrate-producing bacteria are proposed to reduce the risk of CRC mainly via increasing the colonic content of butyrate, which can reduce colonic inflammation and suppress CRC progression.^28–31^

RYGB leads to weight loss mainly through malabsorptive and restrictive mechanisms, whereas VSG is restrictive in nature. RYGB and VSG have been associated with changes in the fecal microbiome, including a possible increased abundance of Proteobacteria, which contain many sulfide-reducing bacterial species.^32^ Recent data have also identified a reduction in fecal butyrate after bariatric surgery; however, data are limited.^33–36^ Therefore, we hypothesized that RYGB and VSG promote a colonic microbial shift that is associated with increased CRC risk, as evidenced by an increase in sulfide-reducing bacteria and reduced butyrate-producing bacteria. Such changes in the host microbiome may offset the benefits of energy restriction in the colon carcinogenesis cascade. To test our hypothesis, we examine the difference in abundance of bacteria and bacterial enzymes involved in sulfide and butyrate metabolism in adults after RYGB or VSG compared to subjects with severe obesity who have yet to get bariatric surgery. To achieve our aims, we implemented a pilot cross-sectional study examining differences in sulfide-reducing and butyrate-producing bacteria and enzymes after RYGB (*n* = 16) or VSG (*n* = 10) compared to pre-surgery (*n* = 15). Our study received Institutional Review Board approval, and patients were seen in the Clinical Research Center by Registered Dietitian Nutritionists trained to collect dietary data. Our post-surgery adults were recruited more than six months after RYGB or VSG to assess their effects on the microbiome in the weight-maintenance phase and after resuming solid foods.^37,38^ We excluded patients if they had other risk factors for colorectal cancer (e.g., family history of CRC); gastrointestinal luminal disease; immunosuppression; bariatric surgery revision; complications after bariatric surgery; psychiatric conditions; or if they had taken weight loss medications, NSAIDs, antibiotics, or probiotics prior to the study endpoint.^1^ Stool samples were collected from patients using the OMNIgene GUT microbiome stool kit. Libraries for shotgun metagenomic sequencing were prepared with the NEBNext Ultra II FS DNA library prep kit according to the manufacturer’s directions. Libraries were then denatured and loaded on the Illumina NovaSeq6000 S4 flow cell at a final concentration of 300pM to generate paired-end 150 bp sequence reads. In our analysis, we focused on differences in DNA abundance of fecal bacteria and enzymes involved in butyrate and sulfide metabolism between pre- and post-bariatric surgery cohorts, quantified using the linear discriminant analysis (LDA) effect size (LEfSe) method. We also validated our findings by performing sensitivity analyses, including an assessment of fecal short chain fatty acids levels.

## Results

**Figure 1.**
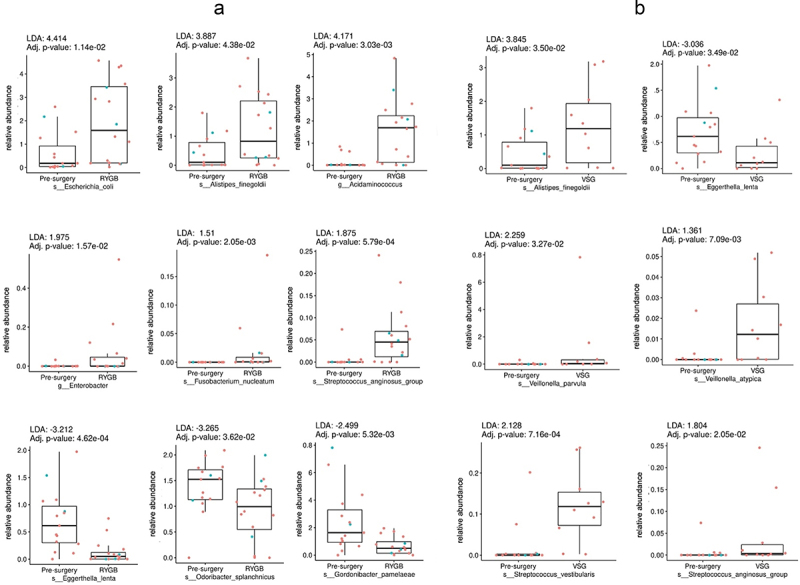
Box plots representing different fecal sulfide-reducing bacteria pre-surgery compared to: (a) Roux-en-Y gastric bypass (RYGB); and (b) vertical sleeve gastrectomy (VSG). The letter s preceding bacteria name indicates it is species and g indicate a genus level. Difference in abundance is measured using linear discriminant analysis (LDA). *P* values adjusted for multiple comparisons (adj. p). Red dots represent females and blue dots, males.

Our post-RYGB and post-VSG subjects were recruited at a median (range) of 24.3 (9.9, 46.5) and 27.0 (18.1, 42.7) months after bariatric surgery, respectively. Median ages were slightly older for the post-RYGB and post-VSG adults than for pre-surgery (48 and 46 vs. 40 years old, respectively). Most participants were female (87%), and the cohort comprised 85% non-Hispanic or Latino white subjects (Table 1). Dietary quality and physical activity trended to be higher after RYGB compared to pre-surgery or VSG. However, all demographics, exercise and dietary variables were not statistically significant when comparing the groups, except for a higher total weight loss post-RYGB than post-VSG (26% vs 19%, *p* < .05). The microbiome data for the RYGB, VSG, and pre-surgery patients are shown in S. Figure 1. There was a greater abundance of Proteobacteria, including *Escherichia coli* and *Klebsiella* in post-RYGB subjects, as well as some Firmicutes (e.g., *Streptococcus* species) and Bacteroides (e.g., *Alistipes finegoldii*) in both post-RYGB and post-VSG cohorts.

**Table 1.** Demographics of included patients’ pre-surgery, post-RYGB and post-VSG.

Variables	Pre-surgery (*n* = 15)^1^	Post-RYGB (*n* = 16)^1^	Post-VSG (*n* = 10)^1^	P-value^2^	
**Age**				0.10	
Median (SD)	40 (9)	48 (8)	46 (14)		
Range	23, 55	29, 57	29, 71		
**Sex**				0.4	
Female	13 (87%)	13 (81%)	10 (100%)		
Male	2 (13%)	3 (19%)	0 (0%)		
**Ethnicity**				0.7	
Hispanic	1 (7%)	3 (19%)	1 (10%)		
NOT Hispanic or Latino	14 (93%)	12 (75%)	9 (90%)		
**Race**				0.12	
Black or African American	5 (33%)	4 (25%)	0 (0%)		
White	10 (67%)	12 (75%)	10 (100%)		
**Months from surgery to fecal collection**					
Median (SD)	NA	24.39 (13.74)	27.02 (8.90)	0.7^3^	
Range	NA	9.99, 46.52	18.18, 42.73		
**BMI pre-surgery**				0.2	
Median (SD)	52 (11)	46 (7)	44 (5)		
Range	34, 72	34, 57	34, 50		
**BMI post-surgery**				0.4^3^	
Median (SD)	NA	33 (7)	34 (4)		
Range	NA	22, 52	28, 40		
TWL% post-surgery*					0.027^3^
Median (SD)	NA	26 (10)	19 (7)		
Range	NA	8, 45	10, 29		
Total exercise (MET minute/week)*				0.8	
Median (SD)	1,980 (3,463)	2,644 (2,798)	1,774 (2,860)		
Range	99, 11988	0, 7,599	0, 9,198		
**HEI Score**				0.2	
Median (SD)	63 (9)	69 (10)	62 (7)		
Range	47, 72	52, 84	55, 77		

^1^n (%).

^2^Kruskal-Wallis rank sum test; Fisher’s exact test.

^3^Wilcoxon rank sum exact test.

*TWL%: Total weight loss percentage, MET: Metabolic Equivalent of Task.

### Fecal abundance of sulfide-reducing bacteria after bariatric surgery

There was a significant difference in the relative abundance of sulfide-reducing bacteria in post-RYGB and post-VSG subjects compared to pre-surgery (LDA detailed in Figure 1). Specifically, there was a higher relative enrichment of fecal *E. coli* (LDA = 4.4, *p* < .05), *A. finegoldii* (LDA = 3.8, *p* < .05), and *Acidaminococcus* (LDA = 4.1, *p* < .05) post-RYGB vs. pre-surgery. *Enterobacter, Streptococcus anginosus* and *Fusobacterium nucleatum* were also more abundant in post-RYGB subjects, but to a lesser extent (LDA below 2, *p* < .05). In contrast, *Eggerthella lenta*, *Odoribacter splanchnicus* and *Gordonibacter pamelaeae* had lower abundances post-RYGB than pre-surgery. Similar to RYGB, the abundance of *E. lenta* was also lower post-VSG when compared to pre-surgery, whereas that of *A. finegoldii* was higher (LDA = 3.8, *p* < .05). Furthermore, post-VSG, the genus *Veillonella* was higher, especially *V. parvula* (LDA = 2.2, *p* < .05). Additionally, sulfide-reducing *Streptococcus* species, specifically *S. vestibularis* (LDA = 2.1, *p* < .05) and *S. anginosus* (LDA = 1.8, *p* < .05), were more abundant in the post-VSG group than in the pre-surgery group.

### Fecal gene abundance of sulfide-metabolizing enzymes after bariatric surgery

**Figure 2.**
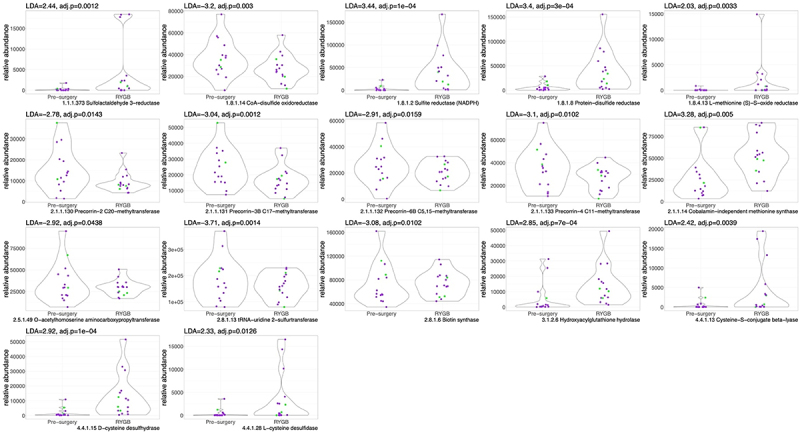
Violin plots for sulfur-related enzyme functional potential (DNA gene copy) after RYGB compared to pre-surgery. Difference in DNA abundance is measured using linear discriminant analysis (LDA). *P* values adjusted for multiple comparisons. Purple dots represent females and green dots, males.

When examining the overall enrichment of enzymes related to sulfide metabolism post-RYGB vs. pre-surgery, we note a statistically significant, higher abundance of enzymes related to sulfur reduction (e.g., EC 1.8.1.2: Sulfite reductase, LDA = 3.4, *p* < .05), methylation (e.g., EC 2.1.1.14: Methionine Synthase, LDA = 3.2, *p* < .05), and transsulfuration pathways (EC 4.4.1.13: Cysteine-S-conjugate beta-lyase, LDA = 2.4, *p* < .05), which are responsible for the production of H_2_S (Figure 2, with detailed description of enzymes in table S3). In contrast, the levels of enzymes involved in B12 synthesis/sulfur oxidation (EC 2.1.1.130–133), biotin synthesis (e.g., EC 2.8.1.6), and sulfur transfer (EC 2.8.1.13) were lower in the post-RYGB group compared to pre-surgery.

**Figure 3.**
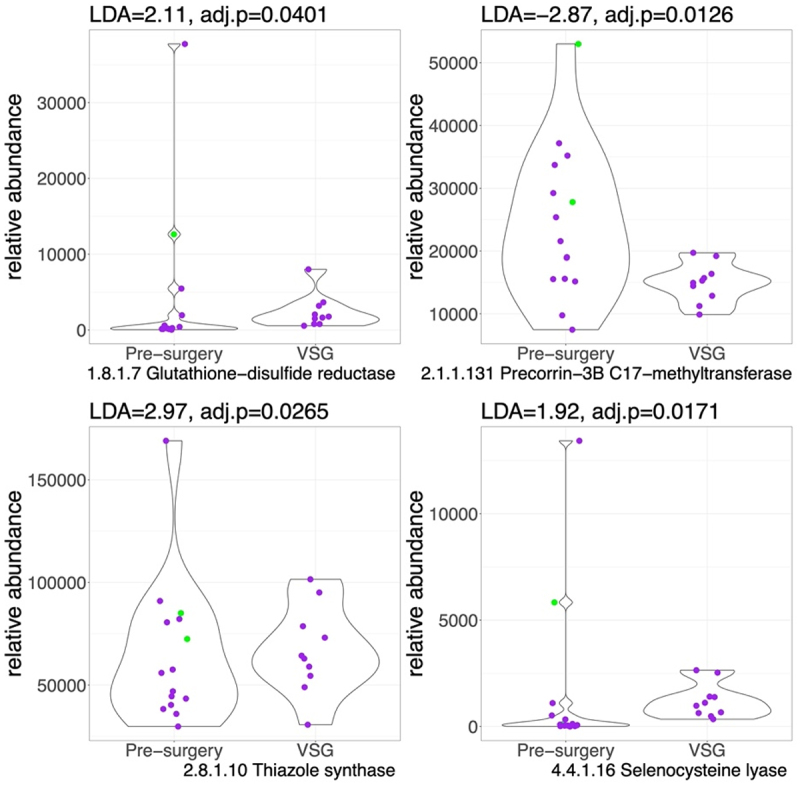
Violin plots for sulfur-related enzyme functional potential (DNA gene copy) after VSG compared to pre-surgery. Difference in DNA abundance is measured using linear discriminant analysis (LDA). *P* values adjusted for multiple comparisons. Purple dots represent females and green dots, males. Outlier values exist pre-surgery, however we still observe a higher abundance of sulfide metabolizing enzymes post-VSG vs. pre-surgery.

Fewer enzymes were differentially abundant after VSG when compared to the pre-surgery cohort (Figure 3). Specifically, enzymes involved in sulfur reduction (EC 1.8.1.7: Glutathione-disulfide reductase, LDA = 2.1; and EC 4.4.1.16: Selenocysteine lyase, LDA = 1.9, *p* < .05), sulfur transfer (2.8.1.10: Thiazole synthase, LDA = 2.9, *p* < .05), were higher post-VSG compared to pre-surgery while enzymes involved in B12 synthesis were lower (EC 2.1.1.131).

### Species-specific differences in sulfide-metabolizing enzymes after bariatric surgery

We further examined the enzymes related to sulfide reduction in specific bacterial species (Table S4). The most pronounced change in RYGB patients was a higher abundance of sulfide-metabolizing enzymes related to *E. coli*. There was also a higher abundance of enzymes found in other sulfide-reducing bacteria, *A. intestini* and *A. finegoldii*, consistent with their higher concentrations in post-RYGB subjects. We also identified a higher abundance of sulfide-metabolizing enzymes for *Klebsiella* in patients post-RYGB. In contrast, there were no differences in the abundance of other sulfide-reducing bacteria (i.e., *Enterobacter, S. anginosus, F. nucleatum, E. lenta, G. pamelaeae*, or *O. splanchnicus*), even though the abundance of these bacteria was significantly altered after RYGB. Similar to RYGB, we identified a higher abundance of sulfide-metabolizing enzymes after VSG that were specific for sulfide-reducing bacteria that were enriched post-VSG (*A. finegoldii*, *S. vestibularis, V. parvula*). Enzymes found in *E. lenta* were lower after VSG, which was consistent with the lower abundance of this species after VSG. Interestingly, the abundance of enzymes found in *A. putredinis* was higher in patients with VSG, even though the relative abundance of this species was not higher after VSG.

### Butyrate producing bacteria and enzymes after bariatric surgery

**Figure 4.**
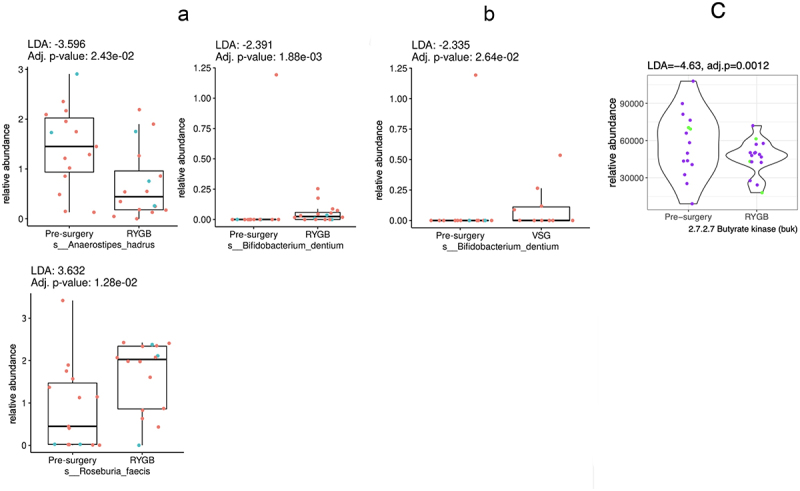
Box plots representing different fecal butyrate-producing bacteria pre-surgery compared to: (a) Roux-en-Y gastric bypass (RYGB); and (b) vertical sleeve gastrectomy (VSG). (c) represents the violin plot for the significantly different butyrate-producing enzyme functional potential (DNA gene copy) after RYGB compared to pre-surgery (no other enzymes were different after RYGB or after VSG). Difference in abundance is measured using linear discriminant analysis (LDA). *P* values adjusted for multiple comparisons. Red or purple dots represent females and blue or green dots, males.

Of the seven butyrate-producing bacteria, three were significantly different in the post-RYGB groups vs. pre-surgery. Specifically, *R. faecis* was more abundant (LDA = 3.6, *p* < .05), whereas *B. dentium* (LDA = −2.3, *p* < .05) and *A. hardus* (LDA = −3.5, *p* < .05) were less abundant post-RYGB, as shown in Figure 4. Only one bacterial species was found to be less abundant after VSG (B. *dentium*, LDA = −2.3, *p* < .05). RYGB was associated with an overall reduction in the abundance of enzymes involved in butyrate production (EC 2.7.2.7, representing butyrate kinase, LDA = −4.6, *p* < .05), as in Figure 4.^39^ No differences were observed in the butyrate-producing enzymes after VSG.

### Sensitivity analyses and the impact of bariatric surgery on the fecal metabolome

We examined the relationship between patient characteristics in Table 1 and microbiome outcomes. In this sensitivity analysis, we did not find an association between our specified bacteria or enzymes and covariates such as age, sex, race, pre-surgery BMI, and the healthy eating index (data not shown). We then assessed whether a lower abundance of butyrate-producing bacteria after bariatric surgery is associated with reduced butyrate and other short-chain fatty acids (SCFA). In this exploratory analysis, we assessed fecal SCFA levels in our cohort using ultra-high performance liquid chromatography – high resolution mass spectrometry (UPLC- HRMS) similar to prior work.^40^ We identify a trend toward lower butyrate and other SCFAs post-RYGB and post-VSG compared to pre-surgery (Table S5). When combining both surgeries, we identify a statistically significant lower total SCFA levels post-bariatric surgery compared to pre-surgery (124.3 vs. 349.2 uMol/g^−1^, respectively, *p* = .01).

## Discussion

Bariatric surgery is an effective long-term weight loss treatment for adults with severe obesity who fail nonsurgical weight loss strategies. Therefore, understanding the health impacts, including the potential cancer-protective effects of bariatric surgery, is critical for this increasingly utilized surgery. Our data add to the existing knowledge by specifically focusing on the impact of bariatric surgery on sulfide- and butyrate-metabolizing bacteria that are increasingly implicated in colorectal carcinogenesis. Our findings identify a higher enrichment of sulfide-reducing bacteria after VSG, but more so after RYGB. Furthermore, reactions involved in sulfur transfer, reduction, and methylation were also enriched in accordance with the higher abundance in sulfide-reducing bacteria after both RYGB and VSG. In contrast, we noted a reduction in the abundance of butyrate-producing bacteria and enzymes after RYGB, which was less pronounced after VSG. These results are consistent with our metabolomic data herein and other studies suggesting a reduction in fecal butyrate after bariatric surgery.^33–36^ Additional, large-scale prospective studies of diverse populations of men and women over the age spectrum are needed to further establish these findings. Furthermore, future studies must address the ability of diet and other strategies to modulate these microbiome changes after bariatric surgery to improve its cancer-protective effects in adults with severe obesity, a cohort with an increased risk of CRC.

Overall, our data support a higher enrichment in pro-carcinogenic sulfide-reducing bacteria and enzymes at more than 6 months post-RYGB and VSG compared to pre-surgery, while butyrate-producing bacteria were lower. Most longitudinal and paired microbiome studies looked at changes within 3–6 months,^41–46^ and up to 12 months after RYGB or VSG.^47–50^ Very few of these studies used whole metagenome sequencing and none of the studies examined paired changes beyond 12 months after bariatric surgery. These paired studies show similar findings to our data with increased Proteobacteria such as *E. coli*,^41–43,45,46,48,50^ *A. finegoldii*,^43,48^ *Fusobacteria*,^42,43,47–49^ *Streptococcus anginosus*^43,48,49^ after RYGB, and increased *V. parvula, A. finegoldii* and *Streptococcus* species VSG with a reduced fermentation efficiency.^43,44^ Similarly, longitudinal studies identify a decrease in *Bifidobacterium* and *Anaerostipes hadrus* after RYGB and VSG.^46,47^ The only studies examining effects beyond one year were cross-sectional like our study and showed similar differences in the microbiome within 8–15 months,^51^ 35 ± 8 months,^49,52^ and 9 years after RYGB when compared to pre-surgery subjects.^34^ In those cross-sectional studies, *Fusobacteriaceae* and *Enterobacteriaceae*, specifically E. coli, were increased after RYGB compared to pre-surgery adults with obesity.^34,49,51^ Furthermore, SCFA such as butyrate decreased after RYGB.^34,49^ Sulfide-reducing bacteria thrive in a protein-rich, low-fiber environment, such as the one provided by the Western diet.^23,25^ In our study, we did not identify an association between the healthy eating index (which includes measures of protein and fiber consumption) and sulfur-metabolizing bacteria after RYGB (data not shown). Our study is, however, too small to clearly define the interactions between surgical weight-loss procedures and associated dietary changes in regard to the structure or function of the colonic microbiome. Therefore, it is probable that the bacterial differences are due to multiple factors including a different energy balance, surgery-induced dietary differences, and the degree of malabsorption and/or maldigestion after RYGB and VSG, which can lead to increased colonic substrates, such as protein, to enrich sulfide-reducing bacteria.^53,54^ Indeed, there is significant malabsorption of fat and protein after RYGB.^55^ Protein maldigestion is also suggested to occur after VSG because of reduced pepsinogen and a major reduction in gastric size.^56,57^ Parallel to a higher abundance of sulfide-metabolizing bacteria, we found fewer butyrate-producing bacteria and enzymes after RYGB which was less evident after VSG. These findings are in accordance with a lower fecal SCFA concentration after bariatric surgery seen in prior studies and in our metabolomic data.^33–36^ We suspect this is related to a reduction in energy intake by approximately 40% after RYGB and lower intake of fiber and starch, which are substrates for butyrate-producing bacteria.^58–67^ Such changes may explain the lower abundance of bacteria and enzymes responsible for butyrate production in the RYGB group. Interestingly, we identify a lesser difference in fermentation efficiency in the post-VSG group, which could be due to the lower degree of energy restriction or a different pattern of dietary intake among those with VSG. Alternatively, our more pronounced findings post-RYGB could be due to the increased colonic oxygen content after RYGB (the air hypothesis), leading to a reduction in strict anaerobes such as butyrate-producing bacteria and an increase in aerotolerant bacteria such as *E. coli*.^68^

### Strengths and limitations

Our study is the first to specifically focus on bacterial metabolic pathways associated with colorectal cancer after bariatric surgery. We report data suggesting differences in the microbiome associated with CRC risk after bariatric surgery. We used careful exclusion criteria to ensure unbiased analysis. Our cross-sectional study was able to investigate a distant timeline at a median 25.6 months after bariatric surgery, while most longitudinal paired pre- and post-surgery studies examined up to 12 months after bariatric surgery.^47–50^ Our findings are also consistent with other longitudinal studies, including the largest paired metagenomic study showing enrichment of *E. coli*, *A. finegoldii*, *Fusobacteria*, *Streptococcus anginosus* after RYGB, and *V. parvula, A. finegoldii* and *Streptococcus* species after VSG.^43^ Our pre- and post-surgery cohorts were also comparable, and sensitivity analysis did not show an association between our specified bacteria or enzymes and covariates such as age, sex, race, pre-surgery BMI, and the healthy eating index (data not shown). Therefore, we suspect that the degree of confounding due to these covariates was small. However, this pilot study was limited by its cross-sectional design and a small sample size, which can undermine power and lead to bias. Our sample size also did not allow for the examination of specific dietary effects or sex differences in sulfide- and butyrate-producing bacteria after bariatric surgery. Unfortunately, most studies examining microbiome changes with bariatric surgery have a small sample size and the biological effects of sex are uncertain.^41,42,44–50^ Finally, it may be more relevant to evaluate the gut microbiome at a later follow-up interval post-surgery since progression to CRC can take 10–15 years. However, the microbiome changes we noted in our findings seem to be similar as early as 3 months and as far as 9 years from bariatric surgery.^34^ This data suggest that the microbiome changes may remain stable later after bariatric surgery and their persistence over a period of years can potentially initiate and/or accelerate colorectal carcinogenesis.

In conclusion, obesity in America remains an epidemic, heralding a parallel rise in bariatric surgery as a treatment option. Obesity is one of the strongest risk factors for CRC. Despite robust metabolic improvement, the impact of bariatric surgery on the risk of CRC is not clear. Our data suggest that the microbiome changes heralded by bariatric surgery may potentially promote CRC risk which warrants further research. In a future follow-up study, we will perform a more rigorous evaluation by looking at longitudinal changes in the same individuals and investigate the potential mechanisms of action by mining microbial biosynthetic gene clusters. Future studies are also needed to examine later changes in the colonic microbiome after surgery, and the impact of these changes on the biomarkers of colorectal cancer risk. Finally, these data provide preliminary information for interventional studies aimed at modulating the microbiome-metabolome axis to improve the risk of CRC after bariatric surgery.

## Patients and methods/materials and methods

### The cohort

The study protocol was approved by the Clinical Scientific Review Committee of the Comprehensive Cancer Center and the Institutional Review Board at The Ohio State University. This cross-sectional study involved 41 patients, of which 15 were pre-surgical and 26 were recruited a median of 25.6 months (range 9.9, 46.5) after surgery (16 RYGB and 10 VSG). Patients were included if they were between the ages 18–75 and were able to voluntarily provide consent. The detailed exclusion criteria are shown in S. Table 1.

### Assessments

Patients were recruited and seen in the Clinical Research Center by Registered Dietitian Nutritionists trained to collect dietary data. The research visit focused on completion of the informed consent form, all baseline questionnaires (demographic factors, medical history, medication use, current health, and physical activity), height and weight, and clinical/laboratory assessments. Additional anthropometric data and weight trajectories after bariatric surgery were collected during the clinic visits. Physical activity was assessed using the international Physical activity Questionnaire, short version (IPAQ).^69^ Participants reported their habitual consumption of food and beverages over 90 days using the online VioScreen Graphical Food Frequency Questionnaire (FFQ, Viocare, Inc., Princeton, NJ, USA).^70^ Overall diet quality was assessed using the Healthy Eating Index-2010 (HEI-2010), which measures compliance with the U.S. Dietary Guidelines for America.^71^

### Fecal sampling

The OMNIgene-GUT stool/feces collection kit (OMR-200, DNA Genotek, Ottawa, Canada) was mailed to patients and returned by mail. The OMNIgene-GUT collection kit was designed for the self-collection of a consistent volume of stool and preservation of microbial DNA. The tube includes a nontoxic stabilizing reagent and a mixing apparatus and is safe for home use. After the sample was collected and the tube was capped, the user vigorously shook the tube for 30 seconds to homogenize and liquefy the sample. At that point, stool DNA would be preserved for at least 60 days at ambient temperature.^72,73^ Patients were instructed on how to complete the collection and mail after completion. The stool samples were stored at −80°C in the laboratories of the investigators for subsequent analyses.

### DNA extraction and metagenomics analysis

DNA extraction was performed using the QIAamp PowerFecal Pro DNA Kit per instruction manual.^74^ Libraries for shotgun metagenomic sequencing were prepared with the NEBNext Ultra II FS DNA library prep kit according to the manufacturer’s directions using the following parameters. The input DNA was 200 ng, followed by a 5-minute fragmentation step. PCR enrichment of the adaptor-ligated DNA was performed with five cycles, and the reactions were purified with 0.6X NEBNext Sample Purification Beads. The final library for each sample was quantified with a fluorometric assay and pooled equally to produce a 1.5 nM final concentration of DNA fragments for sequencing. Libraries were denatured and loaded onto the Illumina NovaSeq6000 S4 flow cell at a final concentration of 300pM to generate paired-end 150 bp sequence reads.

### Sulfide-reducing and butyrate-metabolizing bacteria and enzymes

Our main analysis compared the fecal microbiome of patients with RYGB and VSG to the microbiome preoperatively. Our sulfide-reducing and butyrate-metabolizing bacteria were defined similarly to other studies (Table S2).^22–24,75–81^ Enzymes involved in sulfur and butyrate metabolism and their enzyme commission (EC) numbers were defined as previously described. For the purposes of this project, we have followed the example of Nguyen et al. (2020) and refer to these as sulfide-reducing enzymes.^24^ Enzymes involved in butyrate metabolisms include Butyryl-CoA dehydrogenase (EC 1.3.8.1), Butyrate-acetoacetate CoA-transferase (EC 2.8.3.9), 4-hydroxybutyrate CoA-transferase (EC 2.8.3.-), acetate CoA-transferase (EC 2.8.3.8), and butyrate kinase (EC 2.7.2.7).^82^ Finally, to investigate potential confounders, we assessed associations between other covariates and sulfide-reducing and butyrate-producing bacteria and enzymes.

### The fecal metabolome derivatization, sample preparation, and analyses

The Omnigene kit is comparable to fresh frozen samples for SCFA levels and have been used in prior studies for the measurement of SCFA levels.^83–85^ The SCFA chemical derivatization method was performed as previously reported.^86,87^ Briefly, a mixed standard solution was used for the generation of calibration curve; the solution was first diluted to 20 different concentrations ranging from 4000.00 µM to 0.10 µM. These working standard solutions were prepared before SCFAs derivatization. The concentration and the corresponding peak area were used to generate the calibration curve. Then the standard mixture was diluted using 50% acetonitrile in water. 40 μL standard solution mixed sufficiently with 20 μL of 200 mM 3NPH·HCl solution in a 2 mL Eppendorf tube. Then, 20 μL of 120 mM EDC·HCl-6% pyridine solution was added. Following a 2 min vortex, samples were incubated in a 40°C water bath for 30 min. Samples were cooled down on ice for 1 minute after incubation. The derivatized samples were diluted with 0.92 mL of 10% acetonitrile before UPLC-HRMS analysis. Feces were prepared with a 2:1 propanol ratio (w/w), and then 20 µL of 383 µM ^13^C_4_-sodium butyrate was spiked into these samples and served as internal standards. Before UPLC-HRMS analysis, 60 µL of fecal sample solution was added to 200 µL 50% acetonitrile for SCFAs extraction. After 2 min homogenization, samples were centrifuged at 23,748 × g for 10 min and 40 µL supernatant was used for derivatization following the same procedures described above.

A Thermo Scientific Vanquish Flex UPLC coupled to a Q Exactive™ (QE) mass spectrometer system was used to analyze the derivatized SCFAs. The QE was coupled to a universal ion source with Ion Max heated electrospray ionization (HESI-II) probe. The parallel reaction monitoring (PRM) mode was used for qualitative determination of SCFAs, while targeted selected ion monitoring (t-SIM) mode was used for quantitative analysis. The mass spectra were recorded in negative ion mode. The QE mass parameters were as follows: sheath gas flow rate 10 mL/min, spray voltage 4000 V, capillary temperature 320°C, resolution 70,000 FWHM, Automatic gain control target 5 × 10^4^ (t-SIM), 2 × 10^5^ (PRM), and 1 × 10^6^ (tune method). Separation was performed using a Waters CSH C18 (2.1×100 mm, 1.7 µm) column. The samples were introduced into LC using an auto-sampler (5 µL, maintained at 6°C) in a two-mixture mobile phase: mobile phase A was composed of water (0.1% formic acid), and B was acetonitrile (0.1% formic acid). The initial condition of 15% B held for 2 min and then linearly ramped to 55% B by 9 min, then to 100% B by 0.1 min and held for 1 min. The final value was then decreased linearly to 15% B by 0.1 min and held for 4 min. The mobile phase flow rate was held constant at 0.35 mL min^−1^. The calibration curve, accuracy, precision, recovery and stability of our method were validated according to the FDA guideline for bioanalytical method validation.^88^ Finally, the samples stored in Omnigene stool kit were also assessed for the sulfide metabolome^89^ using our comprehensive metabolomic evaluation platform, but poor signals and large variations were observed so only quantitative SCFA measurements were eventually performed and reported in this study.

### Statistical analysis

Demographics were generated with gtsummary through R and included in Table 1 as medians (Standard Deviation) and ranges. Statistician comparisons were performed, and *p* values were calculated using Fisher’s exact test for categorical variables, Kruskal-Wallis rank sum test for continuous variables across 3 groups, and Wilcoxon test for continuous variables across 2 groups.

Our main microbiome outcomes were for differences in bacteria and bacterial enzymes post-RYGB vs. pre-surgery and post-VSG vs. pre-surgery. The analysis was conducted using a custom-built pipeline. Briefly, raw reads were processed using FASTP.^90^ Reads were then used for taxonomic classification by MetaPhlAn, which uses Bowtie2 to map reads to the CHOCOPhlAn database of predefined clade-specific marker genes from the phylum to species levels.^91^ Output tables were labeled with UniRef90 names for species-level comparison. Bacterial metabolic functions and the abundance of microbial pathways were inferred using HUMAnN3, as described in.^92,93^ The reads were aligned to the reference database UniRef90 (uniref90_201901b_full) provided by HUMAnN. Output tables were labeled with EC category level 4 for enzyme-level comparison. The differences in species abundance and enzyme levels were assessed using LEfSe.^94^ The algorithm explains the differences between classes using the linear discriminant analysis (LDA) effect size (LEfSe) method that combines standard tests for statistical significance with additional tests encoding biological consistency and effect relevance.^94^ p-values were adjusted for multiple hypothesis testing using the Benjamini – Hochberg method. Finally, a Two-sample test was used for comparing post- vs pre- bariatric surgery SCFA levels. Data post-processing and plotting were performed using R programming language.

## Supplementary Material

Supplemental Material

## Data Availability

Our detailed methods are described in detail in this paper. Our investigators will make analytical files available to researchers for noncommercial purposes https://doi.org/10.5281/zenodo.8326652.

## Abbreviations

CRC: Colorectal cancer
EC: Enzyme commission
HEI-2010: Healthy Eating Index-2010
LDA: Linear discriminant analysis
RYGB: Roux-en-Y gastric bypass
SD: Standard Deviation
VSG: Vertical sleeve gastrectomy
