# Supplementary material for: Distinctive patterns of sulfide- and butyrate-metabolizing bacteria after bariatric surgery: potential implications for colorectal cancer risk
Source: Gut Microbes. 2023 Sep 13;15(2):2255345. doi: 10.1080/19490976.2023.2255345 (PMC10501170; doi:10.1080/19490976.2023.2255345)
Supplement: Supplemental Material [file KGMI_A_2255345_SM1673.docx]

**S. Table 1:** Inclusion and exclusion criteria

| Inclusions: |
| --- |
| 1. Aged 18-75 |
| 2. Timepoints: (i) pre-bariatric surgery; or (ii) >6 months and ≤10 years post-VSG or post-RYGB |
| Exclusions: |
| 1.     Are cognitively unable to consent or have physical or mental limitations that would prevent full participation in the study. |
| 2. Have a personal history of precancerous colorectal polyps, colorectal cancer, active malignancy, inflammatory bowel disease, HIV, immune suppression, or cirrhosis. |
| 3.     Have any active GI luminal disease resulting in disturbed gut function, malabsorption, and/or diarrhea. |
| 4. Family history of colorectal cancer in one first-degree or two second-degree relatives, or inherited CRC based on clinical criteria or genetic testing. |
| 5.     Have had more than one previous bariatric surgery. |
| 6.     Have uncontrolled psychiatric conditions at time of enrollment. |
| 7.     Have any known active complication of bariatric surgery (fistula, ulceration). |
| 8.     Take weight loss medications within 1 months of study end point. |
| 9.     Use NSAIDs, antibiotics or probiotics within 3 months of study end point. |

**S. Table 2.** List of sulfide- and butyrate-metabolizing bacteria

| Sulfide reducing bacteria | |  |
| --- | --- | --- |
| Acidaminococcus unclassified | | |
| Adlercreutzia equolifaciens | | |
| Akkermansia | |  |
| Alistipes finegoldii | |  |
| Alistipes putredinis | |  |
| Anaerotruncus colihominis | | |
| Bacteroides clarus | |  |
| Bacteroides intestinalis | |  |
| Bacteroides ovatus | |  |
| Bacteroides plebeius | |  |
| Bacteroides stercoris | |  |
| Bacteroides uniformis | |  |
| Bacteroides vulgatus | |  |
| Bilophila unclassified | |  |
| Bilophila wadsworthia | |  |
| Burkholderiales bacterium | | |
| Campylobacter jejuni | |  |
| Clostridiales bacterium 1 7 47FAA | | |
| Coprococcus catus | |  |
| Desulfobacter | |  |
| Desulfobulbus | |  |
| Desulfotomaculum | |  |
| Desulfovibrio | |  |
| Desulfovibrio desulfuricans | | |
| Escherichia coli | |  |
| Eggerthella lenta | |  |
| Enterobacter | |  |
| Erysipelotrichaceae bacterium | | |
| Erysipelotrichaceae bacterium | | |
| Eubacterium ramulus | |  |
| Eubacterium rectale | |  |
| Fusobacterium nucleatum | | |
| Gordonibacter pamelaeae | | |
| Helicobacter pylori | |  |
| Klebsiella |  |  |
| Lachnospiraceae bacterium 5 1 63FAA | | |
| Leishmania major (protozoa) | | |
| Megasphaera_micronuciformis | | |
| Megasphaera | |  |
| Mycobacterium tuberculosis | | |
| Odoribacter splanchnicus | |  |
| Oxalobacter formigenes | |  |
| Parabacteroides distasonis | | |
| Parabacteroides goldsteinii | | |
| Parabacteroides johnsonii | |  |
| Parabacteroides merdae | |  |
| Parabacteroides unclassified | | |
| Paraprevotella clara | |  |
| Paraprevotella xylaniphila | |  |
| Parasutterella excrementihominis | | |
| Peptococcus niger | |  |
| Prevotella intermedia | |  |
| Roseburia intestinalis | |  |
| Ruminococcus bromii | |  |
| Salmonella thyphimurium | |  |
| Staphylococcus aureus | |  |
| Streptococcus anginosus | |  |
| Streptococcus australis | |  |
| Streptococcus vestibularis | |  |
| Sutterella wadsworthensis | | |
| Veillonella unclassified | |  |
| Veillonella atypica | |  |
| Veillonella parvula | |  |
| Veillonella Dispar | |  |

| **Butyrate producing bacteria** |
| --- |
| Anaerostipes |
| Bifidobacteria |
| Butyricicoccus pullicaecorum |
| Eubacterium |
| Faecalibacterium_prausnitzii |
| Roseburia species (all and subclassification) |

**S. Figure. 1:** Our overall microbiome changes after RYGB (A) and VSG (B) compared to pre-surgery


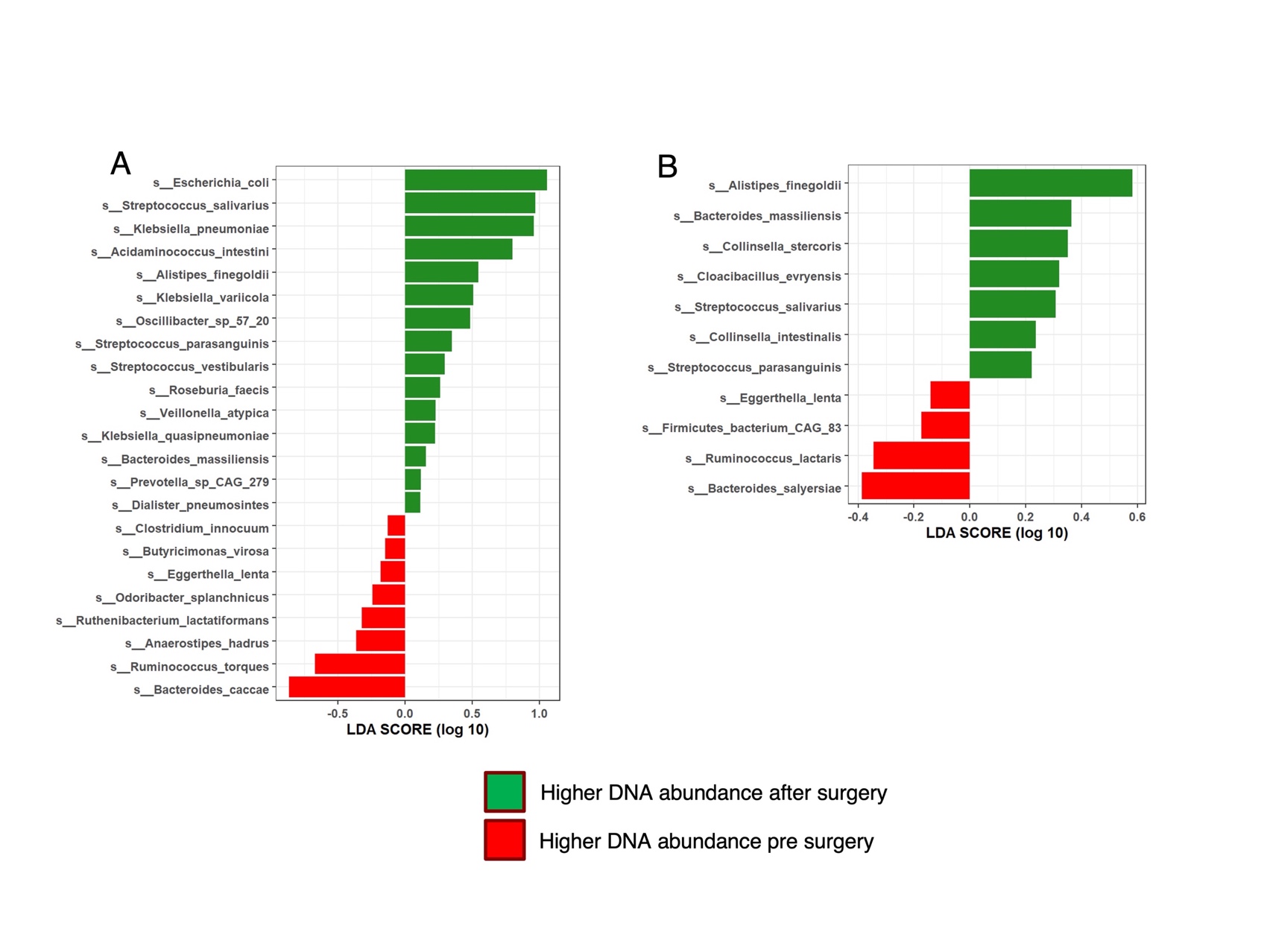


**S. Table 3:** Overall enzymes changes post- vs pre-bariatric surgery.

| Sulfur metabolism | | |
| --- | --- | --- |
| RYGB | | |
| Feature | **Enzyme function** | **Pathway and Description** |
| 1.1.1.373 | Sulfolactaldehyde 3-reductase | Degradation pathway of sulfoquinovose (a major component of organo-sulfur compounds). Specific for E.coli. Involved in the final steps by reducing 2-hydroxy-3-oxopropane-1-sulfonate to 2,3-dihydroxypropane-1-sulfonate |
| 1.8.1.14 | CoA-disulfide oxidoreductase | Oxidizes the sulfur group in two Co-A forming CoA-disulfide, while converting NADP to NADPH. No associated pathway |
| 1.8.1.2 | Sulfite reductase (NADPH) | Sulfite assimilation by reduction of sulfite to sulfide |
| 1.8.1.8 | Protein-disulfide reductase | Reduces a protein disulfide to protein with reduced L-cysteine residues, involves oxidation of NADPH |
| 1.8.4.13 | L-methionine (S)-S-oxide reductase | Cysteine and methionine metabolism. Reduces L-methionine and thioredoxin disulfide to form thioredoxin and Methionine S-oxide |
| 2.1.1.130 | Precorrin-2 C20-methyltransferase | Vitamin B12 metabolism. Oxydation reaction of the sulfur group in S-adenosyl-L-methionine to form S-adenosyl-L-homocystein |
| 2.1.1.131 | Precorrin-3B C17-methyltransferase | Vitamin B12 metabolism. Oxidation reaction of the sulfur group in S-adenosyl-L-methionine to form S-adenosyl-L-homocystein |
| 2.1.1.132 | Precorrin-6B C5,15-methyltransferase (decarboxylating) | Vitamin B12 metabolism. Oxidation reaction of the sulfur group in S-adenosyl-L-methionine to form S-adenosyl-L-homocystein |
| 2.1.1.133 | Precorrin-4 C11-methyltransferase | Vitamin B12 metabolism. Oxidation reaction of the sulfur group in S-adenosyl-L-methionine to form S-adenosyl-L-homocystein |
| 2.1.1.14 | 5-methyltetrahydropteroyltriglutamate-homocysteine S-methyltransferase | Cobalamine-independent Methionine biosythesis from Homcystein. Reaction involves adding a methyl group to the S-group in homocystein |
| 2.5.1.49 | O-acetylhomoserine aminocarboxypropyltransferase | L-homocysteine biosynthesis. Reacts with other thiols and H2S, producing homocysteine or thioethers. An oxidation reaction is involved |
| 2.8.1.13 | tRNA-uridine 2-sulfurtransferase | Protein SAMPylation and SAMP-mediated thiolation. Involved in sulfur transfer. |
| 2.8.1.6 | Biotin synthase | Biotin biosynthesis. Acts on multiple sulfur substrates (dethiobiotin, sulfur-sulfur carrier, S-adenosyl-L-methionine, reduced 2Fe-2S) leading to biotin, sulfur carrier, L-methionine, 5'-deoxyadenosine, oxidized 2Fe-2S. |
| 3.1.2.6 | Hydroxyacylglutathione hydrolase | Methylglyoxal degradation and detoxification to pyruvate. By reducing the sulfur group on S-(2-hydroxyacyl) glutathione, it produces glutathione and a 2-hydroxy carboxylate |
| 4.4.1.13 | Cysteine-S-conjugate beta-lyase | Methionine biosynthesis transsulfuration pathway from O-succinyl-L-homoserine and L-cysteine. Acts on the intermediary compound, Cystathionine, by reducing a sulphur group, which produces L-homocysteine, that is then methylated to methionine |
| 4.4.1.15 | D-cysteine desulfhydrase | Cysteine metabolism. Converts Cystein to H2S and ammonium by reducing a sulphur group. E. Coli specific. |
| 4.4.1.28 | L-cysteine desulfidase | L-cysteine degradation II. Degrades cystein by cleaving and reducing its sulphur group and producing H2S |
| Sulfur metabolism | | |
| VSG | | |
| Feature | **Enzyme function** | **Pathway and Description** |
| 1.8.1.7 | Glutathione-disulfide reductase | Glutathione metabolism. Reduces Glutathione-disulfide to two Glutathiones molecules. |
| 2.1.1.131 | Precorrin-3B C17-methyltransferase | Vitamin B12 metabolism. Oxidation reaction of the sulfur group in S-adenosyl-L-methionine to form S-adenosyl-L-homocystein |
| 2.8.1.10 | Thiazole synthase | Thiamine metabolism. Involved in sulfur transfer. |
| 4.4.1.16 | Selenocysteine lyase | Selenocompound metabolism. Reduces selenocystein to form a selenosulfide (H2Se) and alanine |
| Butyrate metabolism | | |
| RYGB | | |
| Feature | **Enzyme function** | **Pathway and Description** |
| 2.7.2.7 | Butyrate kinase (buk) | Butanoate fermentation. Butyryl-CoA is phosphorylated and transformed to butyrate via butyrate kinase (Buk). . Aso acts, more slowly, on pentanoate and propanoate, and on some branched-chain fatty acids |

**S. Table 4:** Specific changes in enzymes, classified by bacterial species post- vs pre-bariatric surgery.

| Sulfur metabolism | | | | |
| --- | --- | --- | --- | --- |
| RYGB | | | | |
| Feature | **LDA** | **Adjusted P value** | **Enzyme function** | **Pathway and Description** |
| 1.1.1.284_g_Escherichia_s_Escherichia.coli | 2.65394641 | 0.0093687 | S-(hydroxymethyl) glutathione dehydrogenase | Detoxification of formaldehyde by oxydation of glutathione, with generation of NADPH and S-formylglutathione. |
| 1.1.1.373_g_Escherichia_s_Escherichia.coli | 2.21178153 | 0.00965728 | Sulfolactaldehyde 3-reductase | Degradation pathway of sulfoquinovose (a major component of organo-sulfur compounds). Specific for E.coli. Involved in the final steps by reducing 2-hydroxy-3-oxopropane-1-sulfonate to 2,3-dihydroxypropane-1-sulfonate |
| 1.14.14.5_g_Escherichia_s_Escherichia.coli | 2.65317467 | 0.00062897 | Alkanesulfonate monooxygenase | Specific for E.coli that use alkanesulfonates as a sulfur source for growth. The enzyme reduces alkanesulfonate leading to production of sulfite. |
| 1.8.1.2_g_Escherichia_s_Escherichia.coli | 3.16198714 | 0.0005691 | Sulfite reductase (NADPH) | Sulfite assimilation by reduction of sulfite to sulfide |
| 1.8.1.4_g_Alistipes_s_Alistipes.finegoldii | 2.31767193 | 0.01464888 | Dihydrolipoyl dehydrogenase | Glycine biosynthesis I. Oxydizes a sulfur group of donors. An NAD+ oxidoreductase |
| 1.8.1.4_g_Escherichia_s_Escherichia.coli | 2.91968398 | 0.00496409 | Dihydrolipoyl dehydrogenase | Glycine biosynthesis I. Oxydizes a sulfur group of donors. An NAD+ oxidoreductase |
| 1.8.1.7_g_Escherichia_s_Escherichia.coli | 2.85688732 | 0.02160237 | Glutathione-disulfide reductase | Glutathione metabolism. Reduces Glutathione-disulfide to two Glutathiones molecules. |
| 1.8.1.8_g_Escherichia_s_Escherichia.coli | 3.06393918 | 0.00032001 | Protein-disulfide reductase | Reduces a protein disulfide to protein with reduced L-cysteine residues, involves oxidation of NADPH |
| 1.8.1.9_g_Alistipes_s_Alistipes.finegoldii | 2.2183451 | 0.02255537 | Thioredoxin-disulfide reductase | Thioredoxin pathway. Reduces thioredoxin disulfide to thioredoxin |
| 1.8.1.9_g_Escherichia_s_Escherichia.coli | 2.69514586 | 0.0039627 | Thioredoxin-disulfide reductase | Thioredoxin pathway. Reduces thioredoxin disulfide to thioredoxin |
| 1.8.4.11_g_Alistipes_s_Alistipes.finegoldii | 2.18516095 | 0.02566413 | Peptide-methionine (S)-S-oxide reductase | Methionine metabolism. Reduces L-methionine (S)-sulfoxide and oxidizes thioredoxin to form L-methionine and thioredoxin disulfide |
| 1.8.4.11_g_Escherichia_s_Escherichia.coli | 2.61463291 | 0.00099301 | Peptide-methionine (S)-S-oxide reductase | Methionine metabolism. Reduces L-methionine (S)-sulfoxide and oxidizes thioredoxin to form L-methionine and thioredoxin disulfide |
| 1.8.4.12_g_Escherichia_s_Escherichia.coli | 2.6536007 | 0.00087753 | Peptide-methionine (R)-S-oxide reductase | Methionine metabolism. Reduces L-methionine (R)-sulfoxide and oxidizes thioredoxin to form L-methionine and thioredoxin disulfide |
| 1.8.4.14_g_Escherichia_s_Escherichia.coli | 2.41777506 | 0.00072737 | L-methionine (R)-S-oxide reductase | Cysteine and methionine metabolism. Unlike peptide-methionine (R)-S-oxide reductase, this enzyme cannot use peptide-bound methionine (R)-S-oxide as a substrate . |
| 1.8.4.8_g_Escherichia_s_Escherichia.coli | 2.56908067 | 0.0060882 | Phosphoadenylyl-sulfate reductase (thioredoxin) | Assimilatory sulfide reduction pathway. Reduces and assimilates sulfite while reducing thioredoxin disulfide to thioredoxin |
| 1.8.5.3_g_Escherichia_s_Escherichia.coli | 3.05442923 | 0.01011291 | Respiratory dimethylsulfoxide reductase | Electron transfer pathway in Sulfur metabolism. Oxidation of dimethylsulfide to dimethylsulfoxide |
| 2.1.1.13_g_Alistipes_s_Alistipes.finegoldii | 2.27462463 | 0.01635348 | Methionine synthase | Cobalamine-dependent Methionine biosythesis from Homcystein. Reaction involves adding a methyl group to the S-group in homocystein |
| 2.1.1.13_g_Escherichia_s_Escherichia.coli | 2.93088976 | 0.00631571 | Methionine synthase | Cobalamine-dependent Methionine biosythesis from Homcystein. Reaction involves adding a methyl group to the S-group in homocystein |
| 2.1.1.14_g_Acidaminococcus_s_Acidaminococcus.intestini | 2.75927544 | 0.00062203 | 5-methyltetrahydropteroyltriglutamate-homocysteine S-methyltransferase | Cobalamine-independent Methionine biosythesis from Homcystein. Reaction involves adding a methyl group to the S-group in homocystein |
| 2.1.1.14_g_Escherichia_s_Escherichia.coli | 2.57184938 | 0.00072737 | 5-methyltetrahydropteroyltriglutamate-homocysteine S-methyltransferase | Cobalamine-independent Methionine biosythesis from Homcystein. Reaction involves adding a methyl group to the S-group in homocystein |
| 2.3.1.109_g_Escherichia_s_Escherichia.coli | 2.57184938 | 0.00072737 | Arginine N-succinyltransferase | L-arginine degradation II (AST pathway). This pathway converts the carbon skeleton of arginine into glutamate, with the concomitant production of ammonia and conversion of succinyl-CoA into succinate and CoA. |
| 2.3.1.266_g_Acidaminococcus_s_Acidaminococcus.intestini | 2.45224192 | 0.00105076 | [ribosomal protein S18]-alanine N-acetyltransferase. | Reduces the sulfur group in Acetyl-coA to form coA. |
| 2.3.1.266_g_Escherichia_s_Escherichia.coli | 2.41086929 | 0.00010381 | [ribosomal protein S18]-alanine N-acetyltransferase. | Reduces the sulfur group in Acetyl-coA to form coA. |
| 2.5.1.18_g_Escherichia_s_Escherichia.coli | 2.41086929 | 0.00010381 | Glutathione s-transferase | Glutathione-mediated detoxification. Utilizes a gluthathione in the process by transferring a group to sulfide |
| 2.5.1.18_g_Klebsiella_s_Klebsiella.oxytoca | 2.13964181 | 0.0418336 | Glutathione s-transferase | Glutathione-mediated detoxification. Utilizes a gluthathione in the process by transferring a group to sulfide |
| 2.5.1.47_g_Alistipes_s_Alistipes.finegoldii | 2.06212483 | 0.0374517 | Cysteine synthase | L-cysteine biosynthesis from Serin. Utilizes a hydrogen sulfide group and O-acetyl-L-serine to form Cysteine. |
| 2.5.1.47_g_Escherichia_s_Escherichia.coli | 3.06054637 | 0.00087753 | Cysteine synthase | L-cysteine biosynthesis from Serin. Utilizes a hydrogen sulfide group and O-acetyl-L-serine to form Cysteine. |
| 2.5.1.48_g_Escherichia_s_Escherichia.coli | 2.83097659 | 0.00627641 | Cystathionine gamma-synthase | Methionine metabolism. Reacts with hydrogen sulfide and methanethiol as replacing agents, producing homocysteine and methionine, respectively. This reaction leads involves oxidation of a sulfide group. |
| 2.7.7.4_g_Escherichia_s_Escherichia.coli | 3.20201915 | 0.00301866 | Sulfate adenylyltransferase | Sulfide reduction. Produces diphosphate + adenylylsulfate using ATP and Sulfate. Can also produce H2S |
| 2.8.1.1_g_Escherichia_s_Escherichia.coli | 2.66886882 | 0.01667089 | Thiosulfate sulfurtransferase | Sulfide (H2S) oxidation leading to production of thiosulfate. |
| 2.8.1.10_g_Acidaminococcus_s_Acidaminococcus.intestini | 2.44423058 | 0.00105076 | Thiazole synthase | Thiamine metabolism. Involved in sulfur transfer. |
| 2.8.1.10_g_Escherichia_s_Escherichia.coli | 2.77303436 | 0.00154833 | Thiazole synthase | Thiamine metabolism. Involved in sulfur transfer. |
| 2.8.1.12_g_Escherichia_s_Escherichia.coli | 2.65010886 | 0.00049765 | Molybdopterin synthase | Molybdopterin biosynthesis. Involved in sulfur transfer to Molybdopterin. |
| 2.8.1.13_g_Acidaminococcus_s_Acidaminococcus.intestini | 2.42803201 | 0.000903 | tRNA-uridine 2-sulfurtransferase | Protein SAMPylation and SAMP-mediated thiolation. Involved in sulfur transfer. |
| 2.8.1.13_g_Alistipes_s_Alistipes.finegoldii | 2.26035964 | 0.01635348 | tRNA-uridine 2-sulfurtransferase | Protein SAMPylation and SAMP-mediated thiolation. Involved in sulfur transfer. |
| 2.8.1.13_g_Escherichia_s_Escherichia.coli | 2.04142382 | 0.0052045 | tRNA-uridine 2-sulfurtransferase | Protein SAMPylation and SAMP-mediated thiolation. Involved in sulfur transfer. |
| 2.8.1.2_g_Escherichia_s_Escherichia.coli | 2.52467847 | 0.02112716 | Mercaptopyruvate sulfurtransferase | L-cysteine degradation III. Leads to production of hydrogen sulfide (H2S). |
| 2.8.1.4_g_Escherichia_s_Escherichia.coli | 2.84267121 | 0.02030749 | tRNA uracil 4-sulfurtransferase | Thiamine metabolism. Involved in sulfur transfer. |
| 2.8.1.6_g_Acidaminococcus_s_Acidaminococcus.intestini | 2.40282738 | 0.00142463 | Biotin synthase | Biotin biosynthesis. Acts on multiple sulfur substrates (dethiobiotin, sulfur-sulfur carrier, S-adenosyl-L-methionine, reduced 2Fe-2S) leading to biotin, sulfur carrier, L-methionine, 5'-deoxyadenosine, oxidized 2Fe-2S. |
| 2.8.1.6_g_Escherichia_s_Escherichia.coli | 2.77970654 | 0.00297189 | Biotin synthase | Biotin biosynthesis. Acts on multiple sulfur substrates (dethiobiotin, sulfur-sulfur carrier, S-adenosyl-L-methionine, reduced 2Fe-2S) leading to biotin, sulfur carrier, L-methionine, 5'-deoxyadenosine, oxidized 2Fe-2S. |
| 2.8.1.7_g_Acidaminococcus_s_Acidaminococcus.intestini | 2.72410491 | 0.00105076 | Cysteine desulfurase | Cytidylyl molybdenum cofactor sulfurylation, 2Fe-2S iron-sulfur cluster biosynthesis. Removes the sulfur group from Cystein to an S-sulfanyl-acceptor |
| 2.8.1.7_g_Escherichia_s_Escherichia.coli | 2.9878984 | 0.00261042 | Cysteine desulfurase | Cytidylyl molybdenum cofactor sulfurylation, 2Fe-2S iron-sulfur cluster biosynthesis. Removes the sulfur group from Cystein to an S-sulfanyl-acceptor |
| 2.8.1.8_g_Alistipes_s_Alistipes.finegoldii | 2.07528994 | 0.01310289 | Lipoyl synthase | Lipoate (an organosulfur) biosynthesis: Leads to production of sulfide (H2S) |
| 2.8.1.8_g_Escherichia_s_Escherichia.coli | 2.72581913 | 0.00326352 | Lipoyl synthase | Lipoate (an organosulfur) biosynthesis: Leads to production of sulfide (H2S) |
| 3.1.2.12_g_Escherichia_s_Escherichia.coli | 2.76394594 | 0.00153137 | S-formylglutathione hydrolase | Formaldehyde, gluthathione dependent, oxidation (detoxification). Reduces the sulfur group in S-formylglutathione to create glutathione and formate |
| 3.1.2.6_g_Escherichia_s_Escherichia.coli | 2.63508459 | 0.00631571 | Hydroxyacylglutathione hydrolase | Methylglyoxal degradation and detoxification to pyruvate. By reducing the sulfur group on S-(2-hydroxyacyl) glutathione, it produces glutathione and a 2-hydroxy carboxylate |
| 3.1.3.15_g_Alistipes_s_Alistipes.finegoldii | 2.27241769 | 0.02311918 | Histidinol-phosphatase | L-histidine biosynthesis. Reduces the phosphor group in histidinol phosphate leading to its cleavage and creation of phosphate and histidinol. Reason for exclusion: Could not find a relation to sulfur. |
| 3.1.3.15_g_Escherichia_s_Escherichia.coli | 2.30225447 | 0.00712262 | Histidinol-phosphatase | L-histidine biosynthesis. Reduces the phosphor group in histidinol phosphate leading to its cleavage and creation of phosphate and histidinol. Reason for exclusion: Could not find a relation to sulfur. |
| 3.1.6.1_g_Escherichia_s_Escherichia.coli | 2.94592837 | 0.00341084 | Arylsulfatase (type I) | Sphingolipid metabolism, Steroid hormone biosynthesis. Oxidyzes the sulfur group on aryl sulfate substrates leading to production of sulfate |
| 3.1.6.6_g_Escherichia_s_Escherichia.coli | 2.32591693 | 0.00096843 | Choline-sulfatase | Choline-O-sulfate degradation. Acts on Choline sulfate leading to production of sulfite and choline |
| 4.4.1.13_g_Escherichia_s_Escherichia.coli | 2.12999587 | 0.01900373 | Cysteine-S-conjugate beta-lyase | Methionine biosynthesis transsulfuration pathway from O-succinyl-L-homoserine and L-cysteine. Acts on the intermediary compound, Cystathionine, by reducing a sulphur group, which produces L-homocysteine, that is then methylated to methionine |
| 4.4.1.15_g_Escherichia_s_Escherichia.coli | 2.72063111 | 0.00057647 | D-cysteine desulfhydrase | Cysteine metabolism. Converts Cystein to H2S and ammonium by reducing a sulphur group. E. Coli specific. |
| 4.4.1.21_g_Acidaminococcus_s_Acidaminococcus.intestini | 2.40729579 | 0.0008412 | S-ribosylhomocysteine lyase | L-cysteine biosynthesis from L-methionine. Converts S-ribosyl-L-homocysteine to L-homocysteine by reducing a sulfur group. |
| 4.4.1.21_g_Escherichia_s_Escherichia.coli | 2.35252558 | 0.00539009 | S-ribosylhomocysteine lyase | L-cysteine biosynthesis from L-methionine. Converts S-ribosyl-L-homocysteine to L-homocysteine by reducing a sulfur group. |
| 4.4.1.28_g_Escherichia_s_Escherichia.coli | 2.06310389 | 0.01900373 | L-cysteine desulfidase | L-cysteine degradation II. Degrades cystein by cleaving and reducing its sulphur group and producing H2S |
| 4.4.1.5_g_Escherichia_s_Escherichia.coli | 2.61697492 | 0.00976678 | Lactoylglutathione lyase | Methylglyoxal degradation and detoxification to pyruvate (a highly toxic byproduct of glycolysis and fatty acid/ protein metabolism). The enzyme used a methionine molecule that is oxidized in the process of degrading methylglyoxal to S-(2-hydroxyacyl) glutathione |
|  | | | | |
| Sulfur metabolism | | | | |
| VSG | | | | |
| Feature | **LDA** | **Adjusted P value** | **Enzyme function** | **Pathway and Description** |
| 1.8.1.4_g_Streptococcus_s_Streptococcus.vestibularis | 1.218760808 | 0.0017414 | Dihydrolipoyl dehydrogenase | Glycine biosynthesis I. Oxydizes a sulfur group of donors. An NAD+ oxidoreductase |
| 1.8.1.7_g_Streptococcus_s_Streptococcus.vestibularis | 1.138723519 | 0.00045343 | Glutathione-disulfide reductase | Glutathione metabolism. Reduces Glutathione-disulfide to two Glutathiones molecules. |
| 1.8.1.8_g_Alistipes_s_Alistipes.finegoldii | 2.382537584 | 0.02758559 | Protein-disulfide reductase | Reduces a protein disulfide to protein with reduced L-cysteine residues, involves oxidation of NADPH |
| 1.8.1.9_g_Alistipes_s_Alistipes.putredinis | 2.405171943 | 0.04849744 | Thioredoxin-disulfide reductase | Thioredoxin pathway. Reduces thioredoxin disulfide to thioredoxin |
| 1.8.1.9_g_Streptococcus_s_Streptococcus.vestibularis | 1.297903863 | 0.0017414 | Thioredoxin-disulfide reductase | Thioredoxin pathway. Reduces thioredoxin disulfide to thioredoxin |
| 1.8.4.11_g_Alistipes_s_Alistipes.putredinis | 2.366674917 | 0.03434353 | Peptide-methionine (S)-S-oxide reductase | Methionine metabolism. Reduces L-methionine (S)-sulfoxide and oxidizes thioredoxin to form L-methionine and thioredoxin disulfide |
| 1.8.4.11_g_Streptococcus_s_Streptococcus.vestibularis | 1.200966187 | 0.00112848 | Peptide-methionine (S)-S-oxide reductase | Methionine metabolism. Reduces L-methionine (S)-sulfoxide and oxidizes thioredoxin to form L-methionine and thioredoxin disulfide |
| 1.8.4.12_g_Eggerthella_s_Eggerthella.lenta | -1.29593125 | 0.03799979 | Peptide-methionine (R)-S-oxide reductase | Methionine metabolism. Reduces L-methionine (R)-sulfoxide and oxidizes thioredoxin to form L-methionine and thioredoxin disulfide |
| 1.8.4.12_g_Streptococcus_s_Streptococcus.vestibularis | 1.199836194 | 0.00112848 | Peptide-methionine (R)-S-oxide reductase | Methionine metabolism. Reduces L-methionine (R)-sulfoxide and oxidizes thioredoxin to form L-methionine and thioredoxin disulfide |
| 2.1.1.13_g_Alistipes_s_Alistipes.putredinis | 2.369179622 | 0.03974888 | Methionine synthase | Cobalamine-dependent Methionine biosythesis from Homcystein. Reaction involves adding a methyl group to the S-group in homocystein |
| 2.1.1.132_g_Veillonella_s_Veillonella.parvula | 1.046223023 | 0.02708469 | Precorrin-6B C5,15-methyltransferase (decarboxylating) | Vitamin B12 metabolism. Oxidation reaction of the sulfur group in S-adenosyl-L-methionine to form S-adenosyl-L-homocystein |
| 2.5.1.47_g_Alistipes_s_Alistipes.finegoldii | 2.373427251 | 0.04186999 | Cysteine synthase | L-cysteine biosynthesis from Serin. Utilizes a hydrogen sulfide group and O-acetyl-L-serine to form Cysteine. |
| 2.5.1.47_g_Eggerthella_s_Eggerthella.lenta | -1.305491579 | 0.02242632 | Cysteine synthase | L-cysteine biosynthesis from Serin. Utilizes a hydrogen sulfide group and O-acetyl-L-serine to form Cysteine. |
| 2.5.1.47_g_Streptococcus_s_Streptococcus.vestibularis | 1.384512534 | 0.00264816 | Cysteine synthase | L-cysteine biosynthesis from Serin. Utilizes a hydrogen sulfide group and O-acetyl-L-serine to form Cysteine. |
| 2.5.1.47_g_Veillonella_s_Veillonella.parvula | 1.075413923 | 0.02708469 | Cysteine synthase | L-cysteine biosynthesis from Serin. Utilizes a hydrogen sulfide group and O-acetyl-L-serine to form Cysteine. |
| 2.5.1.48_g_Streptococcus_s_Streptococcus.vestibularis | 1.53871852 | 0.00215137 | Cystathionine gamma-synthase | Methionine metabolism. Reacts with hydrogen sulfide and methanethiol as replacing agents, producing homocysteine and methionine, respectively. This reaction leads involves oxidation of a sulfide group. |
| 2.5.1.49_g_Streptococcus_s_Streptococcus.vestibularis | 1.234781676 | 0.00264816 | O-acetylhomoserine aminocarboxypropyltransferase | \| L-homocysteine biosynthesis. Reacts with other thiols and H2S, producing homocysteine or thioethers. An oxidation reaction is involved \| \| --- \| \|  \| |
| 2.8.1.10_g_Eggerthella_s_Eggerthella.lenta | -1.285395824 | 0.03936697 | Thiazole synthase | Thiamine metabolism. Involved in sulfur transfer. |
| 2.8.1.13_g_Streptococcus_s_Streptococcus.vestibularis | 1.535876971 | 0.00090344 | tRNA-uridine 2-sulfurtransferase | Protein SAMPylation and SAMP-mediated thiolation. Involved in sulfur transfer. |
| 2.8.1.4_g_Eggerthella_s_Eggerthella.lenta | -1.285536793 | 0.04968075 | tRNA uracil 4-sulfurtransferase | Thiamine metabolism. Involved in sulfur transfer. |
| 2.8.1.7_g_Streptococcus_s_Streptococcus.vestibularis | 1.364266556 | 0.00264816 | Cysteine desulfurase | Cytidylyl molybdenum cofactor sulfurylation, 2Fe-2S iron-sulfur cluster biosynthesis. Removes the sulfur group from Cystein to an S-sulfanyl-acceptor |
| 2.8.1.7_g_Alistipes_s_Alistipes.putredinis | 2.386492317 | 0.04009338 | Cysteine desulfurase | Cytidylyl molybdenum cofactor sulfurylation, 2Fe-2S iron-sulfur cluster biosynthesis. Removes the sulfur group from Cystein to an S-sulfanyl-acceptor |
| 2.8.1.7_g_Streptococcus_s_Streptococcus.vestibularis | 1.364266556 | 0.00264816 | Cysteine desulfurase | Cytidylyl molybdenum cofactor sulfurylation, 2Fe-2S iron-sulfur cluster biosynthesis. Removes the sulfur group from Cystein to an S-sulfanyl-acceptor |
| 2.8.1.8_g_Alistipes_s_Alistipes.putredinis | 2.376098462 | 0.03500797 | Lipoyl synthase | Lipoate (an organosulfur) biosynthesis: Leads to production of sulfide (H2S) |
|  | | | | |
| Butyrate metabolism | | | | |
| RYGB | | | | |
| Feature | **LDA score** | **Adjusted P value** | **Enzyme function** | **Pathway and Description** |
| 1.3.8.1_g_Anaerostipes_s_Anaerostipes.hadrus | -3.70447 | 0.014255 | Butyryl-CoA dehydrogenase | Rate-limiting step in butyrate production where crotonyl-CoA is transformed to butyryl-CoA |
| 1.3.8.1_g_Roseburia_s_Roseburia.faecis | 3.136501 | 0.023454 | Butyryl-CoA dehydrogenase | Rate-limiting step in butyrate production where crotonyl-CoA is transformed to butyryl-CoA |
| 2.8.3.8_g_Anaerostipes_s_Anaerostipes.hadrus | -3.42896 | 0.009084 | Butyryl CoA:acetate CoA transferase | Butanoate metabolism. Conversts Butyryl-CoA and Acetate to Butyrate and Acetoacetate |

**S. Table 5. Individual short-chain fatty acids (SCFA) levels pre-surgery, post-RYGB and post-VSG and post-surgery (RYGB and VSG). levels are presented as Means and Standard Error of Mean (SEM)**

| SCFA fecal concentrations  (µM/g^-1^) | Pre-surgery | | RYGB | | VSG | | Post-surgery | |
| --- | --- | --- | --- | --- | --- | --- | --- | --- |
|  | Mean | SEM | Mean | SEM | Mean | SEM | Mean | SEM |
| Propionic Acid | 101.88 | 22.99 | 49.18 | 17.94 | 59.11 | 16.09 | 51.35 | 13.66 |
| Butyric Acid | 120.38 | 33.01 | 59.93 | 24.69 | 64.08 | 21.70 | 59.32 | 18.74 |
| Valeric acid | 24.66 | 6.18 | 16.01 | 4.60 | 13.33 | 3.55 | 14.78 | 3.42 |
| Hexanoic acid | 2.59 | 0.84 | 4.84 | 2.81 | 2.32 | 0.65 | 3.87 | 1.96 |
| 2-methylbutyric acid | 40.87 | 12.28 | 14.27 | 3.35 | 19.68 | 5.32 | 15.84 | 3.03 |
| Isobutyric Acid | 37.80 | 12.22 | 12.33 | 3.43 | 15.49 | 4.86 | 13.16 | 2.94 |
| Isovaleric acid | 21.07 | 6.41 | 7.32 | 1.89 | 9.01 | 2.45 | 7.75 | 1.58 |
| Total SCFA* | 349.25 | 90.26 | 163.89 | 54.50 | 183.02 | 51.45 | 124.38 | 33.69 |

*p< 0.05 when comparing pre- and post-surgery for total SCFA.
